# Efficacy and safety of cysto-ventricular catheter implantation for space-occupying cysts arising from glioma and brain metastasis: a retrospective study

**DOI:** 10.1007/s00701-024-05931-4

**Published:** 2024-01-26

**Authors:** Sebastian Niedermeyer, Nicole A. Terpolilli, Pia Nerlinger, Jonathan Weller, Michael Schmutzer-Sondergeld, Stefanie Quach, Niklas Thon

**Affiliations:** Department of Neurosurgery, LMU Hospital, Ludwig-Maximilian-University Munich, Marchioninistrasse 15, 81377 Munich, Germany

**Keywords:** Cysto-ventricular catheter, Minimal-invasive, Space-occupying cysts

## Abstract

**Background:**

Cysto-ventricular catheters (CVC) have emerged as promising treatment option for cystic craniopharyngioma and arachnoid cysts, but their effectiveness in treating cysts originating from glioma or brain metastasis (BM) remains limited. This study aimed to analyze the efficacy of CVC in patients with glioma and BM as well as procedure-associated morbidity.

**Methods:**

This single-center retrospective study included all patients treated with CVC placement for acquired space-occupying cysts deriving from previously treated glioma or BMs between 1/2010 and 12/2021.

**Results:**

A total of 57 patients with a median age of 47 years (IQR 38–63) were identified. Focal neurological deficits were the predominant symptoms in 60% of patients (*n* = 34), followed by cephalgia in 14% (*n* = 8), and epileptic seizures in 21.1% (*n* = 12). Accurate CVC placement was achieved in all but one case requiring revision surgery due to malposition. Three months after CVC implantation, 70% of patients showed symptomatic improvement. Multivariate logistic regression analysis identified the development of space-occupying cysts later in the course of the disease (OR 1.014; *p* = 0.04) and a higher reduction of cyst-volume postoperatively (OR 1.055; *p* = 0.05) were significant predictors of postoperative symptomatic improvement following CVC placement. Local cyst recurrence was observed in three cases during follow-up MRI after an average time of 5 months (range 3–9 months). Further complications included secondary malresorptive hydrocephalus in three cases and meningeosis neoplastica in one patient.

**Conclusions:**

Stereotactic implantation of CVC is an efficient treatment option for patients suffering from symptomatic space-occupying cysts from BMs or glioma, independently from their CNS WHO grade. However, a vigilant approach is crucial regarding potential complications and treatment failures.

## Introduction

In the past decade, overall survival times have increased for various subtyped of glioma and brain metastasis (BM) [7, 19, 25]. However, this improvement in survival has led to a higher likelihood of long-term sequelae [4]. Space-occupying cysts have been reported as complication of glioblastoma treatment as early as 1977, with evacuation considered the most effective treatment method [20]. In the modern era, space-occupying tumor-bed cysts have been reported in 4% of patients after treatment for malignant glioma [3]. After radiosurgery for BMs, intracranial cysts have been observed in 0.9–10% of cases [2, 10, 30]. Similarly, after interstitial brachytherapy (IBT) for the treatment of glioma CNS WHO grade 1, 2, or 3, space-occupying cysts have been reported in 10–12% of cases [22–24]. The occurrence of symptomatic intracranial cysts is even higher, when IBT for glioma CNS WHO grade 1 or 2 is performed during childhood, affecting more than one-third (32.4%) of patients over time [24].

Various surgical options, such as cysto-peritoneal shunting, microsurgical, or endoscopic approaches, have been used to treat acquired symptomatic cysts. While endoscopic surgery or microsurgery are viable techniques in treating arachnoid cysts, they are most successful when the cyst is next to a basal cistern or ventricle, which often is not the case in tumor-associated cysts [6, 18]. Cysto-ventricular catheters (CVC) have proven to be a valuable option for cystic craniopharyngioma and arachnoid cysts [15, 21, 26]. A study by Meissner et al. (2021) reported the results of CVCs in 34 patients with cysts of various entities, including thirteen patients with astrocytoma CNS WHO grade 1–3, one patient with glioblastoma, and two patients with BM [16]. However, since recurrence rate probably also depends on the tendency of the underlying pathology to continuously produce fluid, we focused on cysts from glioma and BMs in the present analysis and excluded cystic craniopharyngioma and non-neoplastic cysts which have been previously analyzed and reported [15, 16, 21]. Therefore, the aim of our current study was to evaluate the efficacy and risk profile of CVC placement in patients with glioma and intracranial metastasis.

## Methods

### Study design, setting, and participants

This retrospective analysis included all consecutive patients with tumor-associated space-occupying intracranial cysts from glioma or BMs who underwent CVC placement at our institution between January 2010 and December 2021. All patients had previously received confirmation of tumor through stereotactic biopsy or resection. Indications for CVC placement were individually defined by our interdisciplinary tumor board and included (a) clinical symptoms related to a space-occupying tumor-related cyst formation, (b) increasing cyst size on follow-up magnetic resonance imaging (MRI), and (3) no radiological signs of solid tumor recurrence or progression necessitating tumor-specific treatment. The diagnosis of space-occupying tumor cysts was confirmed through radiographic assessment. Tumor-associated cysts were defined as an expansive fluid collection in the resection cavity or (in case of other local treatments such as radiotherapy or brachytherapy) in close proximity to a solid tumor. “Space-occupying” was determined based on MRI findings that demonstrated compression of the surrounding structures. Cyst progression on serial imaging was defined as increasing cyst volume on serial cranial imaging.

The study was approved by the independent ethics committee of our medical center (reference no. 22–0511) and was conducted following institutional guidelines. Each patient’s consent to surgery also included consent to the anonymized use of retrospective data for scientific usage.

### Variables, data sources, and measurement

Clinical notes of eligible patients were reviewed for demographic data (gender, age, Karnofsky Performance Status (KPS)), comorbidities, diagnosis, previous treatment, location of the tumor-associated cyst, and preoperative clinical status. Functional assessment included a range of focal deficits, such as motor deficits, sensory deficits, aphasia, apraxia, cerebellar symptoms, and neurocognitive changes, which encompassed alterations in personality and memory. We also considered the presence or frequency of seizures and signs indicative of elevated intracranial pressure, such as headache, nausea, vomiting, and a decreased level of alertness. “Immediate” changes in symptoms or cyst volumes were defined as changes that were registered before discharge from our department. Semiautomatic quantitative volumetry of singular or multiple intracranial cysts was performed on T2-weighted MRI images preoperatively, before discharge, and during follow-up using the SmartBrush tool (*Brainlab*, Munich, Germany).

The primary outcome parameters were the postoperative course of symptoms, cyst volumes, and cyst progression-free survival (cyst PFS), defined as the time after treatment with permanently reduced cyst volume. Patients were categorized as achieving “cyst progression-free survival” when their cyst volume remained consistently reduced within a follow-up of 12 months after the intervention.

### Stereotactic shunt placement technique

**Fig. 1:**
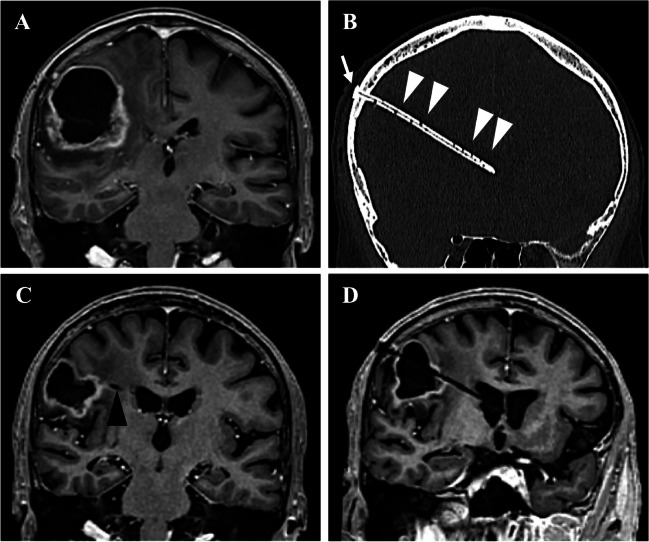
Illustrative case of a 78-year-old patient. Stereotactic biopsy allowed diagnosis of an MGMT-negative glioblastoma located in the right precentral region. Three months after radiation therapy, the patient presented with progressive hemiparesis. **A** T1-weighted Gd-enhanced MRI demonstrated a space-occupying cystic lesion (29.1 cm^3^) with rim enhancement. Stereotactic biopsy excluded tumor recurrency. The patient was scheduled for CVC implantation. **B** Postoperative noncontrast CT-scan in an oblique view showing the entire trajectory of the catheter. The catheter is fixed to the scull with a titanium clip (*white arrow*) and fitted with appropriate holes (*white arrowheads*) allowing drainage of the cyst into the ventricle. **C** T1-weighted Gd-enhanced MRI showing reduction of the cyst (14.9 cm^3^) volume 6 months after CVC placement (*black arrow* indicating CVC). **D** T1-weighted Gd-enhanced MRI in an oblique view showing the trajectory of the catheter

Patients underwent surgery under general anesthesia (total intravenous anesthesia). All surgeries were performed using a frame-based imaging-guided stereotactic technique as previously described [11, 15, 21, 28]. After positioning of the stereotactic frame to the patients’ head, a contrast-enhanced computer tomography (CT, 1-mm slice thickness, 0° tilt) was performed and fused to the preoperatively acquired contrast-enhanced T1 and T2-weighted magnetic resonance images (MRI) as well as a contrast-enhanced MR angiography. For trajectory planning, a software (Brainlab, Munich, Germany) allowing triplanar simulation was used. Stereotactic trajectories were planned either passing through the cystic component into a ventricle or, particularly in case of infratentorial lesions into the basal cisterns, alternatively. After creating a stereotactic localized burr hole, the stereotactic cannula and mandrin were used to perforate the dura mater and reach the target point. The cannula was removed and a vancomycin impregnated ventricular catheter was inserted under stereotactic guidance. Catheter length was determined using the trajectory planning software. A custom made catheter then was produced intra-operatively by fitting the catheter with appropriate holes to allow drainage of the cyst into the ventricle. After insertion, the catheter was fixed on the skull using a titanium clip (Hemoclip, Aesculap, Tuttlingen, Germany). A postoperative CT scan confirmed correct placement of the catheter. An illustrative case is reported in Fig. 1. In patients requiring secondary intraperitoneal CSF diversion, the CVC was exposed on the skull, the titanium clip was removed, and the catheter was connected to an abdominal catheter with interposed adjustable differential pressure unit and gravitational unit.

### Statistical methods

Continuous variables are given as mean ± standard deviation if not indicated otherwise, categorial variables as median ± interquartile range (IQR). Normality testing of preoperative and postoperative cyst volumes was evaluated using D’Agostino & Pearson and Shapiro–Wilk tests, revealing a non-Gaussian distribution of data. We used the Wilcoxon matched-pairs signed rank test to assess differences in cyst volumes before and after treatment. Cyst PFS after CVC placement was analyzed using the Kaplan–Meier method. Univariate and multivariate logistic regressions were performed to identify predictors of postoperative symptomatic improvement. Cox proportional hazard analysis was used to assess whether variables were associated with cyst PFS. For all statistical analysis, a *p*-value < 0.05 was deemed to be significant. All statistical tests were performed with GraphPad Prism 9 (GraphPad Software, La Jolla, CA).

## Results

### Participants and descriptive data

A total of 57 patients were identified (Table 1). The median age of patients was 47 years (IQR 38–63 years). Most of the treated cysts (87.7%) were located in the supratentorial space, while 12.3% were in the infratentorial space. In 66.7% cysts were located in eloquent areas. The distribution of primary tumors was as follows: pilocytic astrocytoma in 12.3% (*n* = 7), astrocytoma, IDH-mutant, CNS WHO grade 2, 3, or 4 in 42.1% (*n* = 24), oligodendroglioma, IDH mutant, 1p/19q-codeleted, CNS WHO grade 2 or 3 in 15.8% (*n* = 9), glioblastoma, IDH-wildtype in 17.5% (*n* = 10), and BMs in 12.3% (*n* = 7).

**Table 1:** Patients’ baseline characteristics

Gender, *n* (%)	Female	27 (47.4)
	Male	30 (52.6)
Age (years), median (IQR)		47 (38–63)
Location	Supratentorial	50 (87.7)
	Infratentorial	7 (12.3)
Histology (CNS WHO grade), *n* (%)	Pilocytic astrocytoma	7 (12.3)
	Astrocytoma (2–4)	24 (42.1)
	Oligodendroglioma (2–3)	9 (15.8)
	GBM	10 (17.5)
	Metastasis	7 (12.3)
Previous therapy, *n* (%)	Resection	24 (42.1)
	Chemotherapy	30 (52.6)
	Radiation	33 (57.9)
	Brachytherapy	29 (50.9)
Time from previous treatment (months), median (IQR)		12 (3–52)
Time from first diagnosis (months), median (IQR)		54 (10–115)

All patients had previously received at least one local (resection, irradiation, interstitial brachytherapy) and/ or systemic (chemotherapy) tumor-specific therapy before CVC implantation (for more details, see Table 1). The median time between the last tumor-specific treatment and CVC placement was 12 months (IQR 3–52 months), and the median time between the first diagnosis of an intracranial tumor and cyst treatment with CVC was 54 months (IQR 10–115 months). The median follow-up after CVC-placement was 18 months (IQR 6–48 months), with 73.2% of patients censored at the time of their last magnetic resonance imaging. Multiple cysts were present in 47% of patients.

### Cyst volume following treatment with CVC

**Fig. 2:**
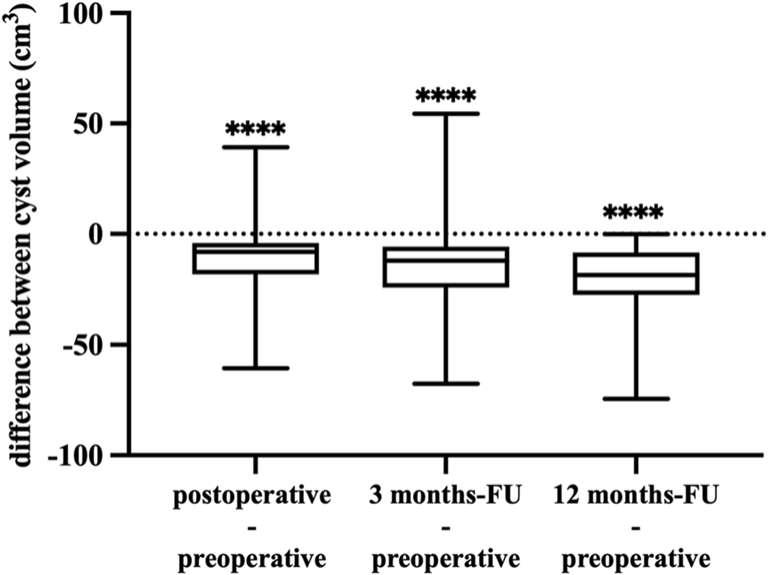
Differences plot illustrating the individual changes in cyst volume before and after treatment at three distinct time intervals. The first box plot on the left reveals the immediate postoperative difference in cyst volume before and after treatment. The second box plot shows difference in cyst volume before treatment and 3 months posttreatment. The third box plot depicts the alterations in cyst volume before treatment and after 12 months following CVC implantation. Comparisons were highly significant (*p* < 0.0001)

**Fig. 3:**
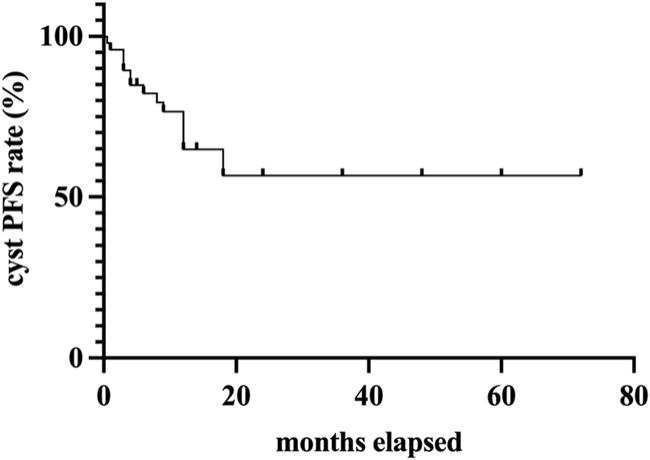
Cyst PFS of patients treated with CVC Kaplan–Meier estimator showing the progression-free survival regarding cyst volume in patients with glioma- and BM-related cysts

Preoperative cyst volume of patients undergoing CVC implantation was 31.5 ml (± 22.65 ml). After CVC placement, there was a reduction in cyst volume to 19 ml (± 16.6 ml), signifying an average reduction of 41%. Individual cyst volume reductions were compared using the Wilcoxon matched-pairs signed rank test, which revealed a significant reduction in cyst volume following treatment (*p* < 0.0001). After a 3-month follow-up period, mean cyst volume decreased to 18.3 ml (± 20.9 ml), corresponding to an average volume reduction of 42%, and 12.9 ml (± 12.9 ml) after 12 months, representing a volume reduction of 59%. In both the 3-month and 12-month follow-up intervals, comparison of cyst volumes with the preoperative baselines demonstrated highly significant differences in both time intervals (*p* < 0.0001) (Fig. 2). The cyst PFS analysis revealed that after 6 months, 69% of patients were still “at risk,” meaning they had not experienced cyst recurrence or been censored. This percentage decreased to 54.2% after 12 months and 27% after 24 months (Fig. 3).

### Surgical procedure and complications

The median surgical time for CVC implantation was 29 min (IQR 23–56 min), and the median hospitalization period (surgery to discharge) was 2 days (IQR 1–2 days). Accurate CVC placement was achieved in all cases, with the exception of one instance of malposition detected in the postoperative CT, necessitating revision surgery. Notably, no other perioperative complications, including hemorrhage, cerebrospinal fluid (CSF) fistula, or local infection, were observed. However, in three cases, secondary dislocation occurred with local cyst recurrence evident on follow-up MRI after an average duration of 5 months (range 3–9 months), indicating that CVC did not result in long-term control for these patients. Additionally, three patients (5.3%) developed communicating hydrocephalus during follow-up, compelling the need for intraperitoneal CSF diversion. Moreover, one patient (1.8%) diagnosed with glioblastoma exhibited signs of meningeosis neoplastica on MRI 3-month post CVC implantation.

### Predictors of clinical improvement at 3-month follow-up

Of treated patients, 24.6% were asymptomatic and underwent treatment due to radiographically determined cyst growth with progressive space-occupying characteristics on MRI. Symptomatic patients with space-occupying cysts presented with cephalgia (15.8%), aphasia (15.8%), motor weakness (29.8%), sensory deficit (15.8%), ataxia (14%), and/ or epileptic seizures (21.1%). One patient presented with dysphagia and dysarthria due to large infratentorial cyst. Median preoperative KPS was 80 (range 50–100). After CVC placement, 67% of symptomatic patients experienced immediate clinical improvement, while symptoms remained unchanged in 33%. Improvement was still observed in 70% and 38.2% of followed-up patients at 3-month and 12-month follow-up, respectively.

Univariate and multivariate logistic regression analysis revealed a statistically significant association of a longer time period from the first diagnosis of the tumor to treatment of space-occupying cysts with symptomatic improvement at 3-month follow-up (OR 1.016; 95%CI 1.006–1.031; *p* = 0.01 and OR 1.014; 95%CI 1.002–1.029; *p* = 0.04, respectively). Additionally, a larger preoperative cyst volume demonstrated a statistical association with clinical improvement (OR 1.033; 95%CI 1.003–1.070; *p* = 0.04). Furthermore, a more substantial reduction in cyst volume following CVC placement showed statistically significant associations with clinical improvement (OR 1.073; 95%CI 1.022–1.052; *p* = 0.02 in univariate logistic regression and OR 1.055; 95%CI 1.01–1.128; *p* = 0.05 in multivariate logistic regression respectively) (Table 2).

**Table 2:** Odds ratio of symptomatic improvement after shunt placement

Variable		Univariate logistic regression			Multivariate logistic regression		
		Odds ratio of symptomatic improvement	95%CI	*p*	Odds ratio of symptomatic improvement	95%CI	*p*
Age		1.012	0.982–1.045	*0.423*			
Gender	Female	2.438	0.816–7.658	*0.115*			
	Male	Referent					
Symptoms	Headache	5	0.799–97.28	*0.146*			
	FND	2.640	0.861–8.406	*0.092*	2.196	0.586–8.731	*0.245*
Cyst location	Supratentorial	1.292	0.233–6.507	*0.754*			
	Infratentorial	Referent					
Histology	Pilocytic astro	0.398	0.071–2.005	*0.262*			
	Astrocytoma	1.889	0.626–6.052	*0.267*			
	Oligodendro-glioma	0.708	0.165–3.196	*0.64*			
	Glioblasotma	0.413	0.0908–1.765	*0.231*			
	Brain metastasis	4.138	0.6389–81.28	*0.204*			
Time between first diagnosis of tumor disease and CVC		1.016	1.006–1.031	0.01*	1.013	1.003–1.027	0.03*
Preoperative cyst volume		1.033	1.003–1.070	0.04*			
Postoperative-preoperative cyst volume		1.073	1.022–1.152	0.02*	1.055	1.010–1.126	0.05*

Odds ratios of symptomatic improvement for different preoperative patient characteristics and cyst-specific variables. (Since preoperative cyst volume and the difference of preoperative-postoperative cyst volume correlated preoperative cyst volume was not included in multivariate logistic regression to increase parsimony). Variables with univariate *p* values ≤ 0.15 underwent multivariate logistic regression

However, age (HR 1.007; 95%CI 0.980–1.036; *p* = 0.609), single cysts (HR 1.025; 95%CI 0.377–2.790; *p* = 0.961), low-grade glioma (HR 0.823; 95%CI 0.259–2.270; *p* = 0.719), high-grade glioma (HR 1.006; 95%CI 0.375–2.819; *p* = 0.990), BMs (HR 1.745; 95%CI 0.270–6.426; *p* = 0.468), or time point of CVC placement after first diagnosis of the tumor disease (HR 0.995; 95%CI 0.986–1.002; *p* = 0.259) did not show a significant association with cyst PFS in univariate cox proportional hazards regression analysis.

## Discussion

The development of space-occupying tumor-associated cysts during the treatment of glioma and BMs can pose significant challenges. However, there is a lack of comprehensive studies on the treatment of intracranial cysts, leaving clinicians with individual decision-making in such cases. Microsurgery and endoscopic surgery have been reported as effective methods for non-neoplastic cysts, but they come with notable short-term morbidity of 27% and 23%, respectively [12, 29].

Another option is shunting, but cystoperitoneal or cystoatrial shunts have inherent complications such as overshunting, valve issues, and infections. Beez et al. reported on 12 patients with space-occupying tumor bed cysts after treatment of high-grade gliomas treated with cystoperitoneal shunting in seven patients or ventriculoperitoneal shunting in case the tumor bed cysts had access to the ventricle [3]. The authors report a clinical benefit in 75% of patients, while nine revision surgeries were necessary in four patients and 33% of patients suffering shunt-related complications [3]. Additionally, there is a risk of tumor-cell seeding in some cases [27]. The overall complication rate for cystoperitoneal shunting in non-neoplastic cysts ranges from 30 to 56% [6, 14]. In light of these complications, cystoventricular catheters have been described to be a viable option for the treatment of cystic craniopharyngioma, arachnoid cysts, and in a mixed cohort of neoplastic and non-neoplastic cysts [15, 16, 21, 26]. The procedure-related morbidity of (stereotactic) CVC placement for different pathologies is low, ranging from 0 to 11%, which makes it an encouraging technique for use in oncological patients [15, 16, 21, 26].

In our study, we contribute our experience with CVC placement for patients with symptomatic or radiologically growing space-occupying cysts. The predominant symptoms in most patients were focal neurological deficits, seizures, and headaches, emphasizing the importance of early imaging in brain tumor patients to identify causative cysts that can be addressed independently of the oncological treatment concept.

Our stereotactic implantation procedure, guided by high-resolution MRI sequences for trajectory planning, resulted in accurate CVC placement in all cases except for one requiring revision due to malposition. The placement of CVCs led to a significant reduction in cyst volume as observed in MRI examinations at discharge, 3-month, and 12-month follow-ups. This reduction was consistently associated with significant clinical improvement, supporting the efficacy of continuous drainage to prevent cyst regrowth, as observed in previous studies on stereotactic single-time cyst aspiration after brachytherapy for low-grade gliomas [13, 16].

While our findings underscore the overall success of CVC placement and perioperative complications such as intracranial hemorrhage, CSF fistula, or local infection were not observed, it is crucial to acknowledge the occurrence of adverse outcomes in 14% (*n* = 8) of patients. Specifically, surgery-associated complications, accounting for 8.7%, included ventricular seeding, malposition, and secondary communicating hydrocephalus. The secondary dislocation of a catheter observed in three patients (5.3%) likely resulted from shrinkage of the drained cyst and progression of an adjacent cyst, indicating treatment failure. The development of communicating hydrocephalus was probably the result of an alteration of CSF dynamics, leading to a reduced resorption and causing hydrocephalus. This is in line with ventricular opening in glioblastoma-surgery being suggested as a risk factor for communicating hydrocephalus [17]. Notably, we adopted a simpler approach to manage hydrocephalus by connecting the CVC to an abdominal catheter, reducing the need for ventricular catheters, and decreasing the morbidity related to VP Shunt placement.

Another critical aspect of cyst drainage is the risk of spreading of tumor cells, exemplified by one case of non-adherent meningeosis neoplastica. The patient underwent CVC placement for the treatment of a lobar tumor-bed cyst after GBM resection and radiotherapy and showed signs of subarachnoid tumor seeding 3 months after surgery. The patient subsequently passed away. While leptomeningeal tumor growth is a typical late-stage manifestation of malignant glioma, it is essential to explore the potential association of CVC placement with the development of meningeosis [1, 8]. Further data is required to accurately estimate the risk of leptomeningeal seeding after CVC placement in glioblastoma patients.

The risk of leptomeningeal metastasis is also a concern in patients with BMs receiving catheter placement. Our study included two patients with BMs from non-small-cell lung cancer and five patients with BMs from breast cancer. The risk of leptomeningeal recurrence is reported to be 3–5% respectively [5, 9]. All seven patients had previously undergone resection, followed by radiotherapy, or radiotherapy alone. At the time of CVC placement, they displayed no signs of local tumor recurrence, thereby reducing the risk of subarachnoid seeding in these patients. The median follow-up for patients with BMs after CVC placement was 6 months (range 1–59 months), and no cases of subarachnoid seeding were observed. Given the absence of larger studies on the risk of ventricular seeding following CVC implantation in patients with treatment-associated cysts stemming from BMs, this treatment should be reserved for patients who have no signs of tumor recurrence or are not suitable candidates for open surgery.

Regression models showed that age and histology of the underlying tumor were not associated with postoperative symptomatic improvement **(**Table 2). Instead, we found an association between of later occurrence of space-occupying cysts with symptomatic improvement (*p* = 0.04). This could indicate that patients with a longer tumor course may better recover from a local pressure effect caused by a cyst formation, possibly because of the more benign tumor disease, affording them the time needed for recovery. Symptoms in those patients may derive from cyst growth rather than other tumor-associated pathologies and therefore making CVC placement more effective. Moreover, our analysis revealed that both a larger preoperative cyst volume and an increased postoperative volume reduction (preoperative-postoperative cyst volume) were significant predictors (*p* = 0.05) of clinical improvement at 3-month-follow-up (Table 2). This finding suggests that larger preoperative cyst volumes with space-occupying radiographic characteristics, as opposed to cysts filling a resection cavity without space-occupying radiographic features, are more likely to benefit from treatment. As highlighted by Steiert et al., following Laplace’ law, the higher surface tension of a space-occupying cyst compared to the ventricle system creates a pressure gradient, leading to a volume shift into the ventricle system, facilitating effective cyst volume reduction, especially in larger and higher-pressure cysts [26]. This mechanism likely contributes to the observed clinical improvement after CVC placement in our study. Taken together, close follow-up of patients with glioma and BMs is crucial to detect and manage space-occupying cysts effectively. CVC placement offers an efficient treatment option, contributing to improved quality of life, particularly in patients with malignant glioma. Nevertheless, further research is needed to better understand the risk of complications and to refine patient selection criteria for optima outcomes.

## Limitations

There are several limitations to our study, first of all its retrospective nature. We also have no comparably sized control group to CVC implantation, which hinders our ability to make direct comparisons. Furthermore, the single-center nature of this study might be influenced by certain treatment strategies that are not directly comparable between different institutions. As with any retrospective study, there is a possibility that unmeasured or unaccounted variables could impact the outcomes. Given the rarity of space-occupying tumor-associated cysts requiring treatment, the limited cohort size compromises the statistical power necessary to detect treatment effects. Consequently, the findings from this study should be regarded as a proof-of-concept. Larger cohort studies will have to provide more robust and generalizable results.

## Conclusion

Stereotactic implantation of CVC is an effective treatment option for patients with space-occupying cysts arising from glioma and BMs. The procedure led to significant cyst volume reduction and symptomatic improvement in the majority of patients. However, vigilance regarding treatment failures and potential complications, including secondary dislocation and aresorptive hydrocephalus or ventricular seeding of tumor cells, is imperative. These findings highlight the importance of close follow-up for patients with glioma and BMs to detect and treat space-occupying cysts, ultimately improving their quality of life. Larger studies are warranted to confirm these results and assess the long-term outcomes.

## Data Availability

Data available on reasonable request.

## Abbreviations

**CVC**: Cysto-ventricular catheter
**Cyst PFS**: Cyst progression-free survival
